# RAFF-AMACNet: Adaptive Multi-Rate Atrous Convolution Network with Residual Attentional Feature Fusion for Satellite Signal Recognition

**DOI:** 10.3390/s25247514

**Published:** 2025-12-10

**Authors:** Leyan Chen, Bo Zang, Yi Zhang, Lin Li, Haitao Wei, Xudong Liu, Meng Wu

**Affiliations:** 1 School of Electronic Engineering, Xidian University, Xi’an 710071, China; cly@stu.xidian.edu.cn (L.C.); yzhang23@stu.xidian.edu.cn (Y.Z.); lilin@xidian.edu.cn (L.L.); 2 China Electronics Technology Group Corporation General Research Institute, Beijing 100041, China; cetc_weiht@139.com (H.W.); liuxudong1986@163.com (X.L.); 3 Science and Technology on Space Physics Technology Laboratory, Beijing 100076, China; wumengnpu@126.com

**Keywords:** satellite signal, automatic modulation recognition, multi-scale feature, atrous convolution, dual-attention collaborative mechanism

## Abstract

With the launch of an increasing number of satellites to establish complex satellite communication networks, automatic modulation recognition (AMR) plays a crucial role in satellite signal recognition and spectrum management. However, most existing AMR models struggle to handle signals in such complex satellite communication environments. Therefore, this paper proposes an adaptive multi-rate atrous convolution network with residual attentional feature fusion (RAFF-AMACNet) that employs the adaptive multi-rate atrous convolution (AMAC) module to adaptively extract and dynamically join more prominent multi-scale features, enhancing the model’s time-series context awareness and generating robust feature maps. On this basis, the pyramid backbone consists of multiple stacked residual attentional feature fusion (RAFF) modules, featuring a dual-attention collaborative mechanism designed to mitigate feature map shifts and increase the separation between feature clusters of different classes under significant Doppler effects and nonlinear influences. On our independently constructed RML24 dataset, a general-purpose dataset tailored for satellite cognitive radio systems, simulation results indicate that at a signal-to-noise ratio of 0 dB, the modulation recognition accuracy reaches 92.99%.

## 1. Introduction

Satellite communication represents a significant achievement that integrates modern communication technology, aerospace technology, and computer technology [1,2,3]. As one of the key enabling technologies, satellite communication plays a significant role in the development of 6G. With 6G progressing from the conceptual stage to pre-standardization and experimental phases, it is expected to extend to higher frequency bands compared to 5G [4]. Meanwhile, both the satellite-ground integrated sensing and communication (ISAC) network [5] and the channel state information (CSI)-based imaging [6] for next-generation 6G heavily rely on research into automatic modulation recognition (AMR) [7].

Traditional AMR approaches can generally be divided into two categories: likelihood-based (LB) methods and feature-based (FB) methods. LB methods [8] determine the modulation type by calculating the likelihood ratio function. However, they necessitate access to the signal and prior knowledge of channel conditions. In non-cooperative communication scenarios, obtaining such prior information is often challenging, and the computation of likelihood functions involving unknown parameters is highly complex. As a result, early AMR research primarily relied on FB methods [9]. In FB methods, manually engineered signal features are used to classify modulation types. For instance, high-order cumulants or constellation diagrams may be extracted as classification features. Obviously, these FB methods require careful design of features tailored to specific signal properties and channel conditions. Therefore, they are not well suited for complex channel environments or scenarios with variable signal-to-noise ratios (SNRs).

With advances in data mining techniques, deep learning (DL) [10] has been increasingly applied to AMR. By integrating feature extractors and classifiers into an end-to-end neural network, DL models can automatically learn high-level features directly from raw signals. Compared to traditional approaches, DL methods do not require extensive prior knowledge or handcrafted features and demonstrate robust modulation recognition performance in complex environments [11,12]. To enhance the ability of deep neural networks to capture more effective and subtle signal features, increasingly complex models have been developed. Among these, network architectures based on multi-scale feature extraction have attracted considerable attention. Standard multi-scale extraction networks are typically categorized into two types: parallel multi-branch architectures and serial skip-connection designs. Both approaches perform feature extraction using different receptive fields to capture signal characteristics at multiple scales. For example, the basic module of the Inception network [13] consists of four parallel branches, which are subsequently combined to form an integrated representation. In contrast, U-Net [14] and Fully Convolutional Networks (FCNs) [15] exemplify serial multi-scale architectures. These models employ skip connections to fuse features from different layers. Parallel structures excel at capturing features with varying receptive fields at the same hierarchical level, while serial structures facilitate the fusion of features across different levels of abstraction. Recent studies have demonstrated that multi-scale networks achieve outstanding performance in fields such as traffic flow prediction [16], robotic machining [17], and aerospace target detection [18]. Therefore, investigating the application of multi-scale networks in satellite communication scenarios has become a current research focus. However, signals in satellite communication systems are affected by long-distance fading [19], nonlinear distortion [20] caused by satellite-borne power amplifiers, and significant Doppler effects [21]. These factors lead to the accumulation of multi-scale feature map shifts across network layers and hinder effective separation of feature clusters, posing a critical challenge. Consequently, previous AMR studies may no longer be directly applicable in this context.

To address these challenges, we propose a simple yet efficient modulation recognition method based on multi-scale feature extraction and fusion, which is suitable for, but not limited to, satellite communication system scenarios. This method mitigates feature map shifts caused by Doppler effects and nonlinear distortion through a designed dual-attention collaborative mechanism. Additionally, it incorporates an adaptive multi-scale feature selection module that enables dynamic extraction of global and local features from various receptive fields, thereby reducing the need for tedious manual tuning. Our contributions of this paper are summarized as follows:

- We design an adaptive multi-rate atrous convolution module with a set of preset dilation rates that adaptively selects and combines features from both global and local contexts to generate robust fused representations.
- A residual attentional feature fusion module based on a dual-attention collaborative mechanism is developed. This module utilizes a channel attention mechanism in the main branch to enhance feature map extraction, while concurrently applying an attentional feature fusion mechanism to integrate features from the skip branch, collaboratively optimizing and compensating for feature map shifts.

Based on the above contributions, the proposed model demonstrates outstanding recognition performance on our self-constructed satellite communication dataset [22]. Further experiments on RML2016.10A [23] and RML2018.01A [24] datasets confirm the generalizability and robustness of the model.

## 2. Related Works

This section is divided into two subsections that respectively review the research status of multi-scale neural networks and AMR tasks within satellite communication systems.

### 2.1. Multi-Scale Neural Networks

Multi-scale neural networks were initially applied primarily in the field of image recognition and have since been extended to various domains, including automatic modulation recognition. For example, Xu et al. [25] proposed a three-stream deep learning framework that inputs both independent and combined in-phase/quadrature (I/Q) components of the signal, extracting and fusing spatial and temporal features across multiple scales. Ref. [26] proposed the use of dilated convolutions combined with a gating mechanism to extract and fuse multi-scale features from remote sensing images, while utilizing skip connections to alleviate training difficulties in the network. Both AMR models proposed in Refs. [27,28] employ multiple parallel dilated convolution modules as multi-scale feature extractors. However, they simply use element-wise addition for fusion, overlooking the imbalance among features at different scales. Ref. [29] proposed a network architecture McNet, which combines several convolutional layers with specific kernels to simultaneously learn spatiotemporal correlations of signals from multi-scale feature maps. Ref. [30] introduced a double-branch multi-scale contextual network that enhances multi-scale feature extraction capability by combining parallel convolutional layers with transformer blocks. It is evident that existing multi-scale neural networks are often accompanied by bulky architectures and high computational complexity. To address this, we optimize the backbone of the multi-scale neural network by adopting a pyramid-like serial structure that extracts feature maps with progressively smaller spatial dimensions while capturing increasingly rich and effective information at each layer.

### 2.2. Modulation Recognition for Satellite Signals

In the modulation recognition task for satellite communication systems, there has been a gradual shift from traditional algorithms based on parameter estimation toward mainstream methods based on deep learning. Ref. [31] developed a low-complexity automatic modulation classification and parameter estimation algorithm based on an analytical study of the Mth-power nonlinear transformation (AMPT). This algorithm is designed to address co-channel interference issues in multi-satellite networks. Jiang et al. [32] proposed a Long-Short Term Deep Neural Network (CLDNN) for satellite signal recognition, which integrates a convolutional neural network (CNN), a long short-term memory network (LSTM), and a deep neural network (DNN), replacing pooling operations with dropout. Ref. [33] demonstrated a transformer-based blind modulation recognition (BMR) algorithm for 6G multibeam mobile satellite (MMS) systems, employing blind and semi-blind channel equalization to overcome challenges such as inter-beam interference (IBI), limited channel-state information, and shadowed-Rician (SR) fading. Ref. [34] proposed a hybrid model combining CNNs and gate recurrent units (GRU), using Doppler frequency shift as the input feature for the neural network. Their results demonstrate that this method performs well under the influence of satellite Doppler shift. Li et al. [35] proposed a novel framework that combines a deep approximate representation module with adaptive feature aggregation, primarily targeting satellite composite modulated signals. However, these methods address only individual challenges within satellite communication systems, lacking a universal neural network architecture for satellite signal recognition. In contrast, we propose a model that combines multi-scale feature extraction with a dual-attention collaborative mechanism, demonstrating strong adaptability and robustness against multiple challenges in satellite communication systems, including Doppler effects, nonlinear distortion, and Rayleigh fading.

## 3. Proposed Methods

In this section, we propose a model called RAFF-AMACNet, which achieves high classification performance using a low-complexity multi-block serial structure. We begin by introducing the satellite communication signal model used for AMR tasks, followed by an overview of the overall architecture of RAFF-AMACNet. Finally, we provide detailed descriptions of each component module within the proposed method.

### 3.1. Signal Model

**Figure 1:**
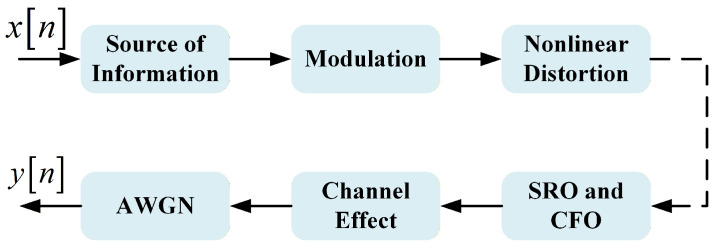
Simplified satellite communication signal propagation process.

Figure 1 depicts a simplified satellite communication system. Before being received by the ground receiver, the satellite signal is influenced by Doppler frequency shift, phase offset, satellite channel effects, amplitude attenuation, and other factors. Specifically, satellites rely on microwave amplifiers capable of sustaining high-power output to achieve ultra-long-distance communication. Compared to power amplifiers used in terrestrial wireless devices, satellite communication systems are more susceptible to significant nonlinear effects. Therefore, unlike conventional terrestrial communication datasets, we explicitly consider the impact of nonlinear distortion caused by satellite-borne power amplifiers on the transmitted signals.

Let y(n) denote the discrete signal received by the ground receiver, which can be mathematically expressed as shown in Equation (2).

(1) $$M\left[n\right]=e^{j\left(2\pi \left(f_{err}\left[n\right]\right)+f_{d}\right)nT_{s}+\varphi \left(\left|s\left[n\right]\right|\right)+\theta_{err}}$$

(2) $$\begin{array}{cc} y\left[n\right] & =G\left(\left|s\left[n\right]\right|\right)M\left[n\right]\sum_{l}h\left[l\right]s\left[n-l-\xi_{err}\right]+z\left[n\right]\\ \; & =y_{I}\left[n\right]+jy_{Q}\left[n\right] \end{array}$$

where $s\left[n\right]$ denotes the transmitted information symbol, $M\left[n\right]$ represents the discrete baseband complex signal, $f_{err}$ indicates the carrier frequency offset (CFO), and $\xi_{err}$ stands for the sampling frequency offset (SFO). Additionally, *h* represents the effect of the satellite channel, and $T_{s}$ is the sampling period. G(∗) and φ(∗) are defined as the amplitude and phase distortions caused by satellite-borne power amplifiers, respectively. We denote $f_{d}$ as the Doppler frequency shift and $\theta_{err}$ as the phase shift introduced during transmission. Finally, $z\left[n\right]$ represents Gaussian white noise. For detailed simulation parameter settings, please refer to Section 4.1 of the paper.

The objective of AMR is to identify the modulation type of the received signal $y\left[n\right]$, thereby providing essential modulation information for subsequent signal demodulation. To standardize the input format for the deep neural network, the in-phase component $y_{I}\left[n\right]$ and the quadrature component $y_{Q}\left[n\right]$ are extracted as discrete real-valued vectors from the complex baseband signal. These components are then normalized separately according to Equation (3).

(3) $$\tilde{y_{i}}=2\times \frac{y_{i}-\min\left(y\right)}{\max\left(y\right)-\min\left(y\right)}-1,$$

Here, y represents either the in-phase or quadrature vector, each containing *L* points, where *L* denotes the number of sampling points per signal. The symbols $y_{i}$ and $\tilde{y_{i}}$ denote the original value and the normalized value at *i*-th sampling point, respectively. Based on this, the vector values are constrained within the range $\left[-1,1\right]$. Subsequently, the two one-dimensional real-valued vectors are concatenated to form a two-dimensional real-valued array (I/Q) of size 2×L. This two-dimensional array serves as the input data format for a single signal sample. Data normalization helps to improve the convergence speed and stability of network training, mitigating the issue of small gradients during parameter updates caused by weak signal amplitude values [36].

### 3.2. The Framework of RAFF-AMACNet

**Figure 2:**
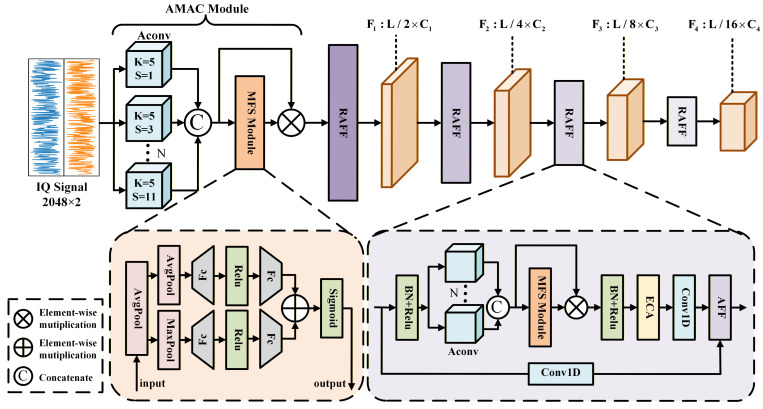
The architecture of RAFF-AMACNet.

The main architecture of the proposed method is illustrated in Figure 2. Table 1 presents the detailed layer-wise parameters of this model.

Specifically, the proposed model consists of three primary stages. In the first stage, we design an adaptive multi-rate atrous convolution (AMAC) module, which employs multiple parallel atrous convolution branches to preliminarily extract multi-scale features from the signal. Additionally, it incorporates a multi-scale feature selection (MFS) module to adaptively weight features at different scales. This module maps the input from a dimension of 2×L to a higher-dimensional space of C×L. The backbone network in the second stage reuses the residual attentional feature fusion (RAFF) module multiple times and employs a multi-layer stacked structure to extract deeper features. Notably, after each RAFF module, the feature length is reduced to half of that in the previous layer. In the final stage of the network, a classification layer is implemented, consisting of a global average pooling (GAP) layer followed by a fully connected (FC) layer. Due to its simple structure, the classification layer is shown in Table 1.

From an overall architecture perspective, the entire network can be regarded as hierarchical adaptive receptive field modeling combined with multi-scale feature recalibration. The AMAC module explicitly constructs multiple receptive fields, and the MFS module learns the weights of different scale branches, thereby producing a set of sample-adaptively recalibrated multi-scale fused features. Subsequently, the stacked multi-layer RAFF modules, leveraging the integrated dual-attention collaborative mechanism, progressively aggregate low-level features from shallow layers into high-level features that represent global temporal dependencies. Therefore, during training, the network can adaptively extract local texture details from small receptive fields and global envelope features from large receptive fields, respectively, and after multiple layers of iteration, output deep features that are beneficial for classification tasks.

#### 3.2.1. Adaptive Multi-Rate Atrous Convolution Module (AMAC)

Unlike most multi-scale networks that utilize parallel convolutional structures, the core concept of the adaptive multi-rate atrous convolution (AMAC) module is to employ a set of atrous convolutions [37] as feature extractors, followed by a multi-scale feature selection (MFS) module. This design enables the neural network to adaptively assign more effective weights to relevant features while suppressing redundant feature channels. Such a unique approach eliminates the need to manually design different convolution receptive field sizes at each layer, thereby saving considerable manual effort.

Atrous convolution is a generalized convolution operator that introduces a dilation factor into the sampling pattern of the input feature map. Standard implementations keep a dense kernel and modify the indices at which input values are sampled, effectively skipping a fixed number of positions between adjacent kernel elements. The dilation rate controls this sampling pattern and thereby enlarges the receptive field of the convolution without increasing the number of learnable parameters or reducing the resolution of the feature maps. With a fixed kernel size, employing multiple atrous convolutions with different dilation rates enables the network to aggregate multi-scale contextual information from the input feature maps, which is particularly beneficial for capturing long-range dependencies in the data. Leveraging the advantages of atrous convolutions, we integrated them as feature extraction units within the AMAC module.

The AMAC module employs multiple parallel atrous convolution branches, each with distinct dilation rates, to process the input and extract multi-scale features. Atrous convolutions have a larger receptive field compared to traditional convolutions and can more effectively capture spatial structures and detailed information within satellite signals. First, we preset a set of dilation rates for atrous convolutions, $d=\left\{1,3,5,7,9,11\right\}$, to construct multi-scale spatial receptive fields. Using a combination of odd-numbered dilation rates helps avoid overlapping sampling positions and reduces sampling discontinuities caused by the gridding effect. This approach enhances the diversity and uniformity of the receptive field. Each branch uses a fixed kernel size of 5, striking a balance between model sensitivity to local details and computational efficiency. Excessively large kernel sizes not only significantly increase computational burden but may also weaken the model’s ability to capture fine-grained local features. The input $X\in {ℜ}^{B\times C\times L}$ is processed through multiple parallel atrous convolution branches to obtain feature maps $x_{i}\in {ℜ}^{B\times C\times 1\times L}$ for each scale, where i=1,2,…,N. Here, *B* denotes the batch size, *C* is the number of channels, *L* represents the signal length, and *N* denotes the number of convolution branches. If we follow the traditional design approach of multi-scale networks, it is necessary to manually set different dilation rates at each layer to achieve optimal performance. To overcome this limitation, we design the MFS module, illustrated in Figure 2, to eliminate manual intervention, enabling the network to adaptively emphasize important scale features. At the beginning of this module, to compress the channel information of each receptive field while preserving the temporal structure of the features, we first apply average pooling along the channel dimension *C* to integrate the initial feature $X_{concat}\in {ℜ}^{B\times C\times N\times L}$ into a unified representation for each atrous convolution branch. Subsequently, average pooling and max pooling operations on the two branches are performed to capture the mean response ${\tilde{F}}_{Avg}\in {ℜ}^{B\times 1\times N\times 1}$ and the salient response ${\tilde{F}}_{Max}\in {ℜ}^{B\times 1\times N\times 1}$ along the sequence dimension *L*, respectively. The scale dimension *N* is then processed through two fully connected layers, enabling the model to adaptively generate weight representations at each scale. At the end of the MFS module, a sigmoid activation function maps the combined values between 0 and 1, producing the multi-scale weight matrix $M_{ms}\in {ℜ}^{B\times 1\times N\times 1}$ that we need. The computation formulas are provided below:

(4) $$\begin{array}{cc} {\tilde{F}}_{Avg} & =AvgPool\left(AvgPool\left(X_{concat}\right)\right)\\ & =\frac{1}{L\times C}\sum_{i=1}^{L}\sum_{j=1}^{C}X_{concat} \end{array}$$

(5) $$\begin{array}{cc} {\tilde{F}}_{Max} & =MaxPool\left(AvgPool\left(X_{concat}\right)\right)\\ \; & =\max_{i=1,2,..,L}\left(\frac{1}{C}\sum_{j=1}^{C}X_{concat}\right) \end{array}$$

(6) Mms=SigmoidMLP2ReluMLP1F˜Avg⊕MLP4ReluMLP3F˜Max=σW2δW1F˜Avg⊕W4δW3F˜Max

(7) $${\tilde{X}}_{concat}=Flatten\left(M_{ms}⊗X_{concat}\right)$$

Here, *C* refers to the number of input channels, and *N* denotes the number of atrous convolution branches. The functions σ and δ correspond to the sigmoid and ReLU nonlinear activation functions, respectively. The weight vectors $W_{1},W_{2},W_{3},W_{4}$ belong to the fully connected layers. ⊕ indicates element-wise addition, while ⊗ represents the Hadamard product (element-wise multiplication). By computing the formulas given above, we obtain the multi-scale weight matrix. Finally, the weight matrix is multiplied element-wise with the feature $X_{concat}$ and then flattened to produce the weighted multi-scale fused feature ${\tilde{X}}_{concat}\in {ℜ}^{B\times \left(C\times N\right)\times L}$.

#### 3.2.2. Residual Attentional Feature Fusion Module (RAFF)

We found that applying residual connections via simple addition after the attention mechanism generally does not improve model performance. This traditional approach tends to exacerbate feature map misalignment and blur the boundaries of feature clusters corresponding to different modulation types, particularly under severe Doppler frequency shifts and nonlinear effects in satellite communication scenarios. Consequently, the dual-attention collaborative mechanism is designed to address this issue and is embedded within the residual attentional feature fusion (RAFF) module, as illustrated in Figure 2.

**Figure 3:**
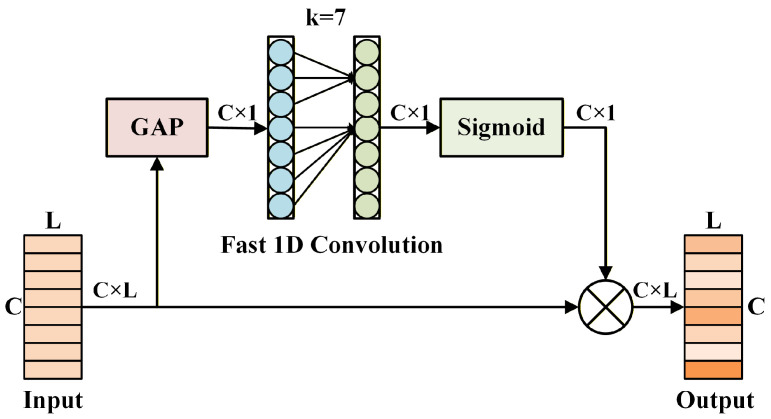
The architecture of the ECA module.

Each RAFF module contains an embedded AMAC module to further extract multi-scale information from input signals at every layer. Additionally, batch normalization and ReLU activation layers are positioned at the beginning and middle of the RAFF module. This arrangement helps reduce the risk of overfitting and accelerates training convergence by normalizing the input data distribution. After extracting the multi-scale features, it is important to recognize that each channel’s feature maps contribute differently to the classification results. To address this, we employ the efficient channel attention (ECA) [38] to apply attention weighting along the channel dimension of the high-dimensional features. The ECA structure is illustrated in Figure 3.

ECA is an enhanced variant of the squeeze-and-excitation (SE) attention mechanism [39]. It substitutes the fully connected layers in SE with a fast 1D convolution of size *k*. This convolution operation has a strong capability to capture cross-channel information, enabling the network to learn the importance of each channel’s features. By adjusting the output weights of various channels, the network can more efficiently focus on features that are most beneficial for the current task. The specific computation of ECA is detailed in Equation (8).

(8) $$\begin{array}{cc} \tilde{X} & =X⊗Sigmoid\left(Conv1d\left(AvgPool\left(X\right)\right)\right)\\ \; & =X⊗\left(\sigma \left(W\left(X_{Avg}\right)+b\right)\right) \end{array}$$

Here, $X\in {ℜ}^{B\times C\times L}$ represents the input feature, σ denotes the sigmoid activation function, and $AvgPool\left(\cdot \right)$ is the average pooling operation across the channel dimension. *W* and *b* refer to the weights and biases of the convolution layer, respectively, and ⊗ denotes element-wise multiplication. Then, a one-dimensional convolution is applied after the ECA module to perform multi-scale feature mixing and pooling. The stride and kernel size of this convolution are set to 2 and 3, respectively, reducing the output feature length to half of the input length. Notably, an additional one-dimensional convolution is applied on the skip branch to match the shapes of the output and input feature maps.

Furthermore, to address the issues mentioned at the beginning of this subsection, particular attention must be given to the fusion method between the deep features processed by the ECA module and the shallow features from the skip branch. We find that the attentional feature fusion (AFF) module [40] effectively strengthens the correlation between imbalanced features across both spatial and channel dimensions, facilitating finer-grained feature refinement.

**Figure 4:**
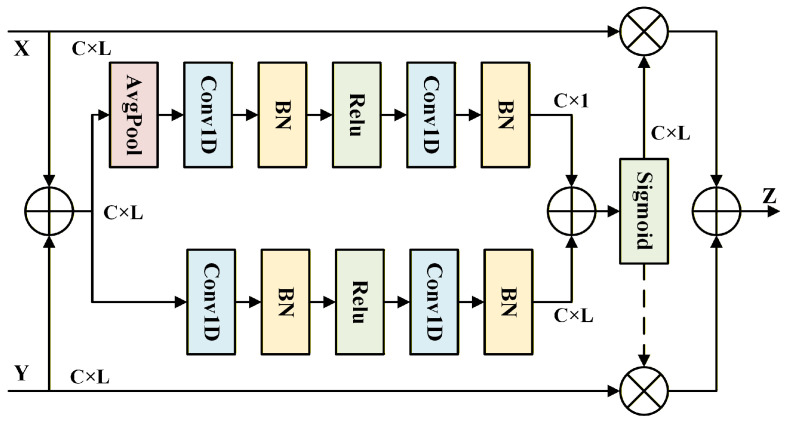
The architecture of the AFF module.

The core purpose of this module is to fuse two features from different levels. To achieve this, two branches are designed to extract the global and local information of these features. First, as shown in Figure 4, for acquiring local information $W_{L}\in {ℜ}^{B\times C\times L}$, the inputs *X* and $Y\in {ℜ}^{B\times C\times L}$ are combined by element-wise addition and then fed into a branch composed of convolutional layers, batch normalization (BN) layers, and activation layers. Unlike the local branch, the global branch additionally applies an average pooling operation to the initially fused features, enabling the model to focus on the features from a global perspective and produce a global information output $W_{G}\in {ℜ}^{B\times C\times 1}$. Finally, $W_{G}$ is broadcasted and added to $W_{L}$, and a sigmoid activation function is applied to generate the attention weights $W_{deep}\in {ℜ}^{B\times C\times L}$.

The output of the AFF module is calculated as follows:

(9) $$W_{G}=W_{2}\left(\delta \left(W_{1}\left(AvgPool\left(X⊕Y\right)\right)+b_{1}\right)\right)+b_{2}$$

(10) $$W_{L}=W_{4}\left(\delta \left(W_{3}\left(X⊕Y\right)+b_{3}\right)\right)+b_{4}$$

(11) $$W_{deep}=\sigma \left(W_{G}⊕W_{L}\right)$$

(12) $$Z=\left(W_{deep}⊗X\right)⊕\left(\left(1-W_{deep}\right)⊗Y\right)$$

where *Z* denotes the output feature produced by the AFF module, while *X* and *Y* represent the deep and shallow features. $W_{deep}$ denotes the attention-based weighting factor applied to the deep features.

However, the deep feature *X* input to the AFF module itself requires more refined and discriminative channel information. Otherwise, redundant and weakly responsive channels may hinder the learning of fusion weights, leading to error accumulation. This approach, which we term the dual-attention collaborative mechanism, has been integrated into our proposed module. The dual-attention collaborative mechanism is a generalized description of the combined use of ECA and AFF. By combining the two, the network incorporates attention mechanisms both during the feature enhancement stage and the feature fusion stage, enabling the model to adapt to both global channel weights and local structural information simultaneously.

## 4. Experiments

In this section, we present a series of experiments to demonstrate the overall superiority of the proposed model. These experiments are conducted on the satellite communication dataset RML24 [22] and extended to two widely used public datasets: RML2016.10A [23] and RML2018.01A [24]. Since this study primarily focuses on the satellite communication scenario, the experimental results and analysis mainly pertain to the RML24 dataset. The other two datasets are only used to present experimental results for validating the model’s robustness. Considering practical deployment scenarios, we primarily report the number of model parameters, the floating point operations (FLOPs), and the inference time per sample to analyze the computational complexity. Following standard evaluation protocols for AMR tasks, we report three metrics: overall accuracy (OA), Macro-F1 score, and Cohen’s Kappa coefficient. The computational formulas for these evaluation metrics are provided below:

(13) $$OA=\frac{\sum_{i=1}^{N}TP_{i}+\sum_{i=1}^{N}TN_{i}}{\sum_{i=1}^{N}TP_{i}+\sum_{i=1}^{N}TN_{i}+\sum_{i=1}^{N}FP_{i}+\sum_{i=1}^{N}FN_{i}}$$

(14) $$Precision=\frac{\sum_{i=1}^{N}TP_{i}}{\sum_{i=1}^{N}TP_{i}+\sum_{i=1}^{N}FP_{i}}$$

(15) $$Recall=\frac{\sum_{i=1}^{N}TP_{i}}{\sum_{i=1}^{N}TP_{i}+\sum_{i=1}^{N}FN_{i}}$$

(16) $$Macro-F1=\frac{2\times Precision\times Recall}{Precision+Recall}$$

(17) $$Kappa=\frac{p_{o}-p_{e}}{1-p_{e}},p_{e}=\frac{\sum_{i=1}^{N}a_{i}\times b_{i}}{N\times N}$$

where *N* denotes the total number of modulation types, $TP_{i}$ represents the number of correctly predicted positive samples for the *i*-th class. $FP_{i}$ indicates the number of falsely predicted positive samples for the *i*-th class. $TN_{i}$ is the number of correctly predicted negative samples for the *i*-th class. $FN_{i}$ denotes the number of falsely predicted negative samples for the *i*-th class. $p_{o}$ is numerically equivalent to the OA. $a_{i}$ and $b_{i}$ represent the actual and predicted number of samples for each class, respectively.

### 4.1. Datasets

**RML24:** Unlike publicly available datasets, it is specifically constructed for satellite communication applications. The main simulation parameters are as follows: the sampling rate is 1 MHz; the number of samples per sampling period is set to 5; the maximum frequency deviations for SRO and CFO are 50 Hz and 500 Hz, respectively; the satellite-borne power amplifier is modeled using the Saleh model; the single-sample Doppler frequency shift is randomly sampled within the range [−1 KHz, 1 KHz]; and the phase shift is randomly sampled within $\left[0,2\pi \right]$. The modulation types include BPSK, QPSK, OQPSK, SOQPSK-TG, FQPSK, ARTM, 8PSK, GMSK, 16QAM, 32QAM, and 64QAM. The SNR ranges from −20 dB to 20 dB in steps of 2 dB. In total, the dataset comprises 231,000 samples, with 1000 samples per modulation type at each SNR level. Each signal sample consists of 2048 points. More detailed parameter settings of RML24 are provided in Ref. [22], and the dataset is publicly available at https://terabox.com/s/1uT2uewv2kcMEjgjlTTimBw (accessed on 21 June 2025).

**RML2016.10A:** This dataset [23] is generated using GNU Radio and contains a total of 220,000 signal samples. The SNR ranges from −20 dB to 18 dB in steps of 2 dB. It includes 11 modulation types: BPSK, QPSK, 8PSK, 16QAM, 64QAM, PAM4, CPFSK, GFSK, WBFM, AM-SSB, and AM-DSB. For each modulation type and SNR level, 1000 sample instances are provided. All signals are uniformly sampled with 128 data points per sample.

**RML2018.01A:** Compared to earlier datasets, this dataset [24] introduces higher-order modulation. It comprises 24 distinct types of analog and digital modulations, including BPSK, QPSK, 8PSK, 16PSK, 32PSK, 16APSK, 32APSK, 64APSK, 128APSK, 4ASK, 8ASK, 16QAM, 32QAM, 64QAM, 128QAM, 256QAM, FM, GMSK, OQPSK, AM-SSB-SC, AM-DSB-SC, AM-SSB-WC, AM-DSB-WC, and OOK. The SNR ranges from −20 dB to 30 dB in steps of 2 dB. For each modulation type and SNR level, 4096 signal samples are provided, with each sample consisting of 1024 points.

### 4.2. Experimental Setup

All experiments are conducted using the PyTorch (version 2.5.0) deep learning platform. Network training is performed on an NVIDIA RTX 5000 Ada GPU. The training process encompasses 100 epochs, starting with a learning rate of 0.001. Additionally, the dataset is randomly split into training, validation, and testing sets with a ratio of 8:1:1. All samples in these subsets are generated from different signal sources and are mutually independent; the batch size is set to 32; and the Adam optimizer is employed for network training. The AMAC module consists of six parallel atrous convolution branches with dilation rates of $d=\left\{1,3,5,7,9,11\right\}$. All atrous convolution kernels in the AMAC module are fixed at a size of 5. The ECA module uses a convolutional kernel size of 7, and the upsampling rate in the AFF module is set to 2. The RAFF module is composed of 4 stacked layers, with output channel dimensions configured as 30, 60, 60, and 60 for each layer.

### 4.3. Comparisons with Other Methods

We evaluate RAFF-AMACNet against seven representative baseline models for AMR tasks, including CNN-based models (CNN4 [41], MCNet [29], ResNet [42], and AWN [43]) and hybrid CNN-LSTM models (CLDNN [32], MCLDNN [25], and PETCGDNN [44]). Among these models, CNN-based models are proficient at local spatial modeling and capturing the texture features of signals; however, their ability to model long-term temporal dependencies is limited, making it challenging to extract robust features in highly dynamic satellite environments. Hybrid CNN-LSTM models are better suited for processing sequential data and can more effectively extract the temporal characteristics of satellite communication signals, but they lack the capability to capture fine-grained texture details, particularly the envelope and phase variations induced by nonlinear effects.

Table 2 provides a detailed comparison of the model parameter size and commonly used evaluation metrics across all models on the three datasets. Focusing on the RML24 dataset, RAFF-AMACNet achieves the best performance across all three metrics. Specifically, the proposed method attains the highest OA of 71.74%. Compared to the worst-performing model, CNN4, this represents improvements of 17.95% in OA, 19.22% in Macro-F1, and 19.73% in Kappa. Even compared to the CLDNN model, which is well-suited for sequence processing, RAFF-AMACNet outperforms it by approximately 5.9% across all three evaluation metrics. Applying the recent state-of-the-art (SOTA) model AWN to the AMR task within the context of satellite communications, it achieves an OA of 64.87%. While this is higher than that of most baseline models, it remains nearly 7% lower than that of the proposed model. In addition, in terms of model parameter size, PETCGDNN has the fewest parameters at 75.91K, but its OA reaches only 63.89%. Our proposed model slightly increases the number of parameters while achieving significantly higher recognition performance.

The computational complexity of the baseline and proposed models can be analyzed using the FLOPs and inference times reported in Table 2. The following primarily focuses on comparing the computational complexity of each model on the RML24 dataset. Among all models, MCNet has the lowest FLOPs, yet its inference time is not the shortest. This is primarily due to the extensive use of small convolutional kernels, multi-branch structures, and frequent tensor concatenation operations in MCNet, which reduce hardware parallelization efficiency and consequently increase the actual inference time. In contrast, PETCGDNN benefits from its simple architecture and the use of GRU, achieving an inference time of only 0.043 ms per sample. Furthermore, the proposed model exhibits a longer inference time than PETCGDNN because the AMAC module contains multiple dilated convolution branches, which are processed sequentially during the forward pass. Overall, with an appropriate number of parameters and computational complexity, the proposed model achieves the best recognition performance.

Table 3 presents OA of all models on the RML24 across varying SNR levels. When the SNR drops below −6 dB, the recognition performance of all models deteriorates significantly due to the dominant noise, resulting in poor classification accuracy. Within the SNR range of −6 dB to 0 dB, where noise levels remain high, RAFF-AMACNet demonstrates a notable improvement in classification performance, achieving an OA of 70.63% at −6 dB and reaching 92.99% at 0 dB. Although MCNet achieves a recognition rate of 17.36% at an SNR of −18 dB, outperforming RAFF-AMACNet by approximately 1.7%, its performance at other SNR levels is markedly inferior to that of the proposed model. In addition, the recent SOTA model AWN benefits from its integrated adaptive wavelet decomposition module, achieving strong recognition performance at SNRs above 6 dB and reaching a peak accuracy of 98.66%. However, once the SNR drops below 6 dB, its performance deteriorates markedly. This degradation occurs because the combined effects of noise and nonlinear distortion severely contaminate the local frequency and envelope features extracted by the wavelet decomposition, preventing the model from capturing meaningful representations. Overall, when the SNR exceeds −12 dB, the proposed model consistently maintains a significant advantage over all baseline models. This clearly demonstrates that RAFF-AMACNet, employing its novel multi-scale feature extraction and fusion strategy, efficiently captures high-dimensional features of various modulation types, even from signals corrupted by high levels of noise. These results underscore the model’s robust feature extraction capability and superior noise resilience, highlighting its suitability for deployment in complex electromagnetic environments.

**Figure 5:**
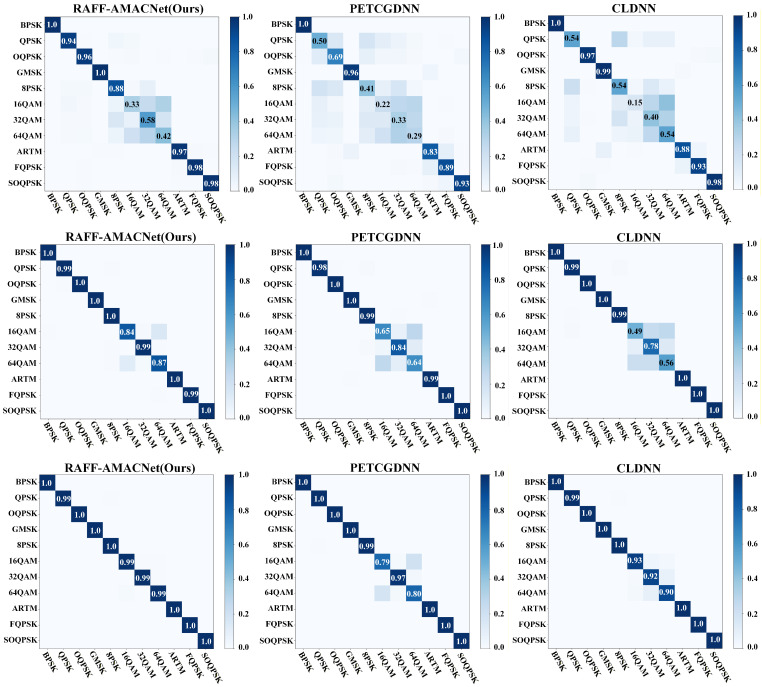
Confusion matrices of three models with outstanding classification performance on the RML24 dataset—RAFF-AMACNet, PETCGDNN, and CLDNN. The first row corresponds to an SNR of −4 dB, the second row to 4 dB, and the third row to 12 dB.

Figure 5 presents the confusion matrices of three models illustrating their classification performance. At a low SNR of −4 dB, all three models exhibit notable misclassification errors for QAM modulation classes. However, at SNR levels of 4 dB and 12 dB, the proposed method significantly outperforms the others by effectively distinguishing between different QAM modulation types. Notably, at an SNR of 12 dB, the classification accuracy for nearly all modulation types approaches 100%. In contrast, PETCGDNN and CLDNN continue to exhibit a certain degree of confusion among QAM classes, even under high-SNR conditions. This indicates that the proposed model is capable of extracting effective multi-scale features starting at a signal-to-noise ratio of −4 dB. Additionally, leveraging the dual-attention collaborative mechanism, the model effectively optimizes and compensates for feature map shifts caused by satellite Doppler effects and nonlinear distortion. For QAM modulation signals, it successfully separates feature clusters in high-dimensional space, increasing the spatial distance between different modulation types and thereby achieving accurate classification.

To thoroughly analyze the recognition performance of the proposed model for each category, Table 4 presents three metrics (precision, recall, and Macro-F1) at an SNR of −4 dB for each class. It can be observed that the three metrics for most modulation types exceed 90%, indicating that the model can perform classification in a stable and robust manner. However, at an SNR of −4 dB, the primary performance limitations occur in the recognition of 16QAM, 32QAM, and 64QAM modulation types. The recall for 16QAM is only 33.01%, indicating that the model missed many samples of this class. Furthermore, the precision for 32QAM suggests that the model misclassified a large number of samples from this category as other classes. Below, we discuss the outstanding cluster separation capability demonstrated by the proposed model at sufficiently high SNRs, especially for the QAM categories.

**Figure 6:**
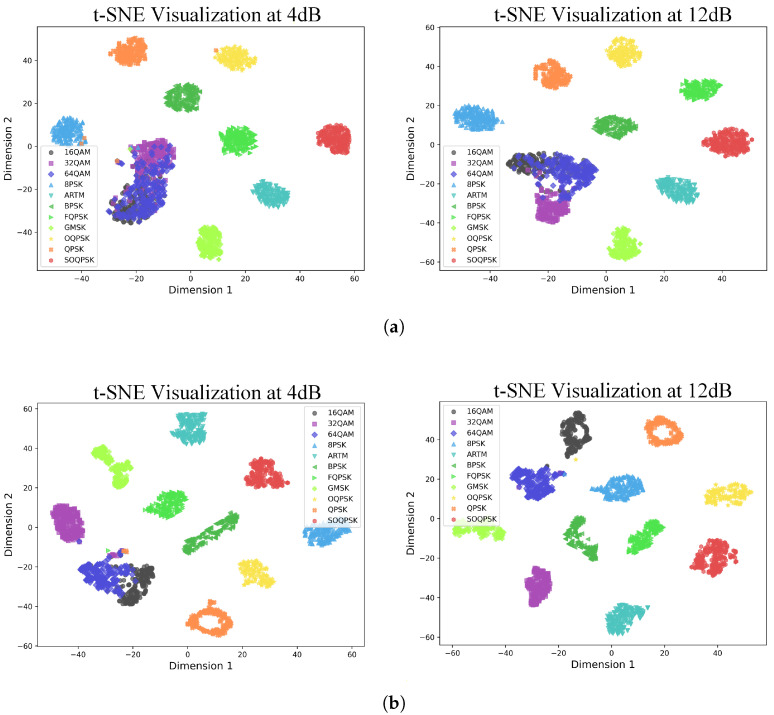
Feature distribution visualization before the classifier via t-SNE at 4 dB (**left**) and 12 dB (**right**) SNR. (**a**) The CLDNN model. (**b**) The proposed model.

As shown in Figure 6, the CLDNN model, which achieves the best performance among the baseline models, still produces severe aliasing in the features of QAM modulation at an SNR of 4 dB. This aliasing impedes the model’s ability to accurately classify this modulation type. Specifically, compared to other modulation types, QAM modulation exhibits densely packed constellation points with multiple amplitude and phase levels. In satellite communication scenarios, Doppler effects and nonlinear distortions blur symbol mappings, causing distortion and dispersion of constellation points, with the most significant impact on QAM modulation signals. In deep learning-based modulation recognition tasks, such signal distortions manifest as feature map shifts and ineffective cluster separation. The proposed model addresses this issue. At an SNR of 4 dB, the 32QAM feature clusters are completely separated from other QAM modulation clusters, while the 16QAM and 64QAM clusters exhibit only slight overlap. Even at a high SNR of 12 dB, the CLDNN model still exhibits blurred boundaries for some feature clusters, whereas the proposed model is capable of clearly separating the feature clusters of all modulation types with distinct boundaries.

**Figure 7:**
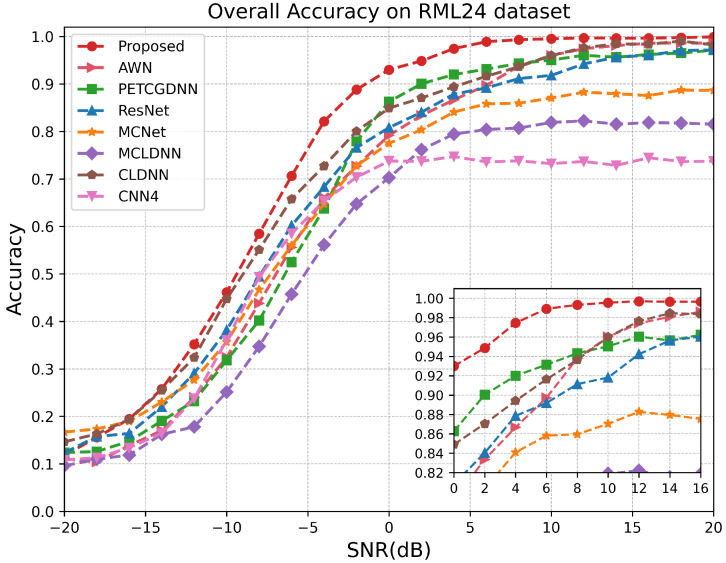
Test accuracy comparison of different models over different SNR levels on RML24.

**Figure 8:**
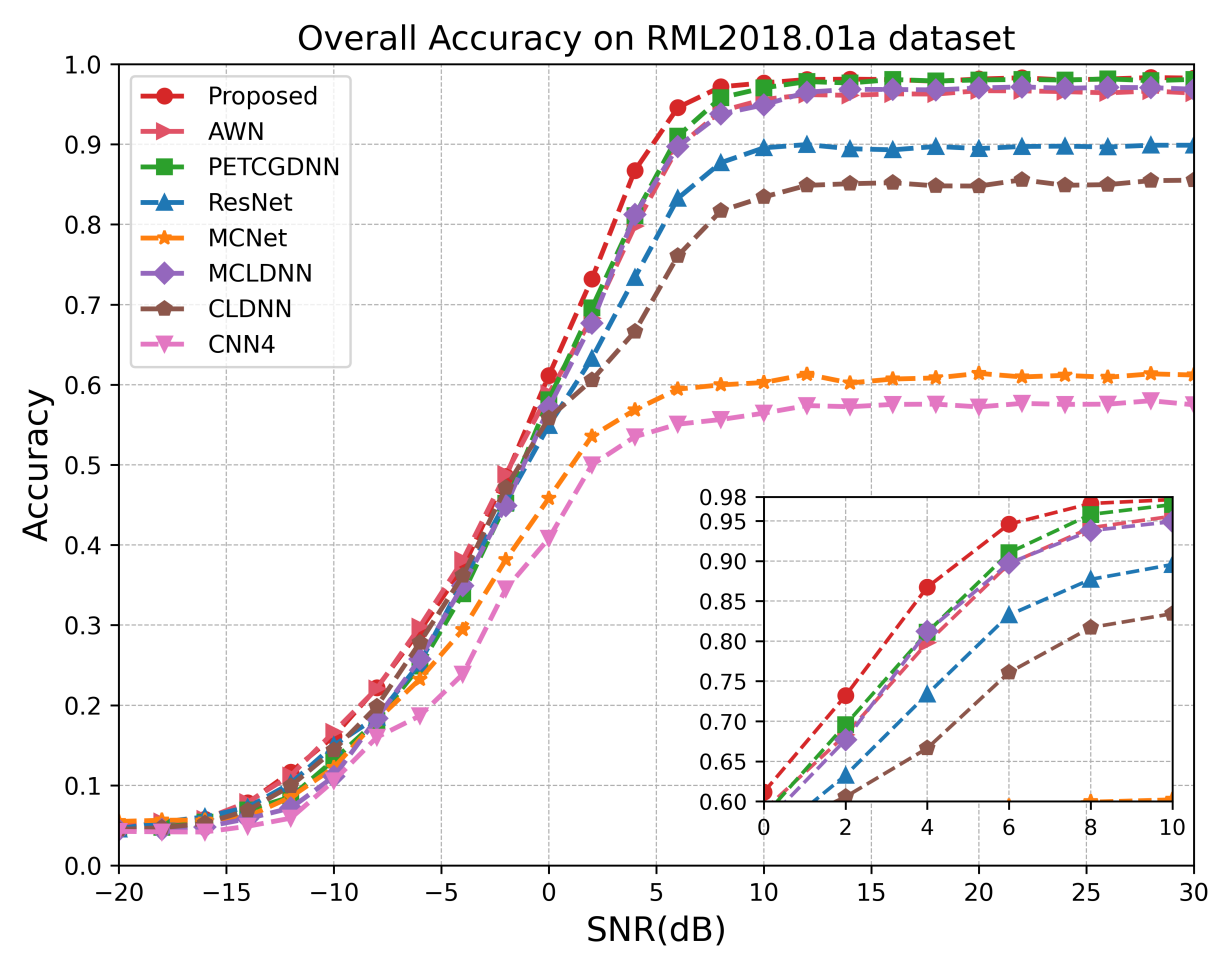
Test accuracy comparison of different models over different SNR levels on RML2018.01A.

**Figure 9:**
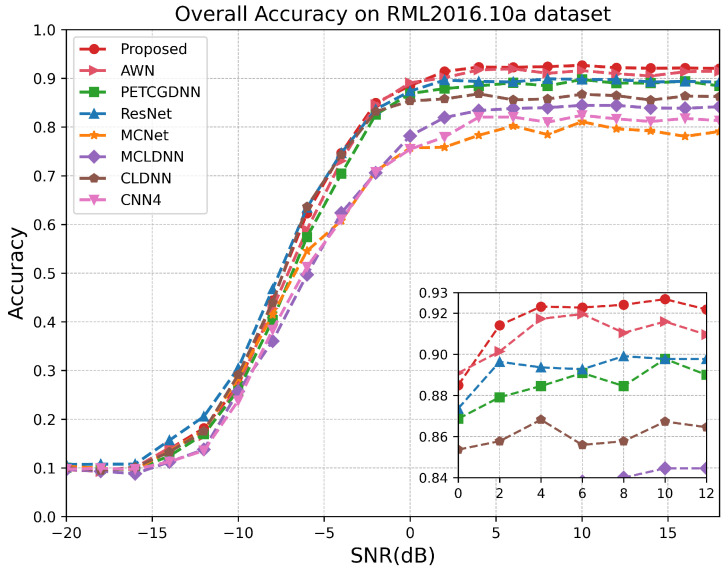
Test accuracy comparison of different models over different SNR levels on RML2016.10A.

As illustrated in Figure 7, on the RML24 dataset, the proposed model demonstrates superior performance for SNRs above −10 dB, particularly within the range of −6 dB to 10 dB. Since the proposed model comprehensively extracts effective features from both local and global perspectives of the signal and employs a dual-attention mechanism to fuse multi-scale information, it achieves a recognition accuracy exceeding 99% at sufficiently high SNRs. Notably, when the SNR exceeds 14 dB, the recognition performances of the CLDNN and AWN models closely approximate that of the proposed model. This is because, at high SNRs, the temporal structure of satellite signals remains largely intact and is minimally corrupted by noise, allowing the LSTM or wavelet decomposition module to effectively extract stable sequential features. Figure 8 demonstrates that within the SNR range of 0 dB to 6 dB on the RML2018.01A dataset, our model exhibits a significant advantage over other methods. According to Figure 9, it can be seen that on the RML2016.10A dataset, when the SNR is below −8 dB RAFF-AMACNet performs slightly worse than some baseline methods. Once the SNR exceeds 0 dB, the advantage of RAFF-AMACNet begins to emerge.

Considering the results across the three datasets, the proposed model demonstrates the most significant performance improvement on RML24, while the enhancement on the public datasets is relatively limited. This is because, based on the characteristics of the AMAC module, the proposed model can better adapt to signals with longer sampling points and satellite communication scenarios by extracting richer global–local features at multiple scales. Conversely, when the number of sampling points is relatively small, such as 1024 or 128, this advantage is constrained by the signal length. More importantly, to maintain experimental consistency and focus on the primary objective, we fixed the hyperparameters finely tuned on the RML24 dataset. This may also have limited the proposed model’s performance on public datasets from being fully demonstrated. Even so, these results still highlight the superiority, generalizability, and robustness of RAFF-AMACNet in handling various AMR task scenarios, including but not limited to satellite communication environments.

### 4.4. Ablation Study

**Figure 10:**
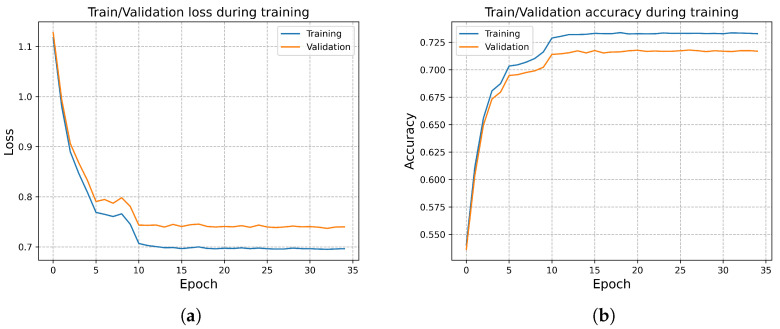
(**a**) Training and validation loss curves. (**b**) Training and validation accuracy curves.

At the beginning of this section, we present the training and validation loss curves of the proposed model on the RML24 dataset. As shown in Figure 10, no overfitting is observed throughout the training process. Thanks to data normalization, the network exhibits fast convergence and high stability. Subsequently, we conduct ablation experiments on RML24 to evaluate the complexity and necessity of each module within RAFF-AMACNet.

Table 5 summarizes the five model variants constructed for ablation purposes, each incorporating different combinations of the proposed components. Specifically, $Model_{0}$ represents the baseline network, consisting solely of the AMAC module while replacing the MFS module with a simple concatenation operation; $Model_{1}$ integrates the MFS module into the network; $Model_{2}$ adds the ECA module within the RAFF module; $Model_{3}$ integrates only the AFF module within the RAFF module; and $Model_{4}$ includes all modules—AMAC (MFS) and RAFF (ECA and AFF). As shown in Table 5, the complete model outperforms the other four variants in overall classification performance. Due to the adaptive enhancement of multi-scale features by the MFS module, $Model_{1}$ achieves a 2.03% improvement in the OA metric compared to the baseline model. This demonstrates the necessity of integrating the MFS module within the AMAC module. By comparing the results of $Model_{1}$, $Model_{2}$, $Model_{3}$, and $Model_{4}$, we find that neither $Model_{2}$ and $Model_{3}$ lead to an improvement in OA. This is primarily because introducing only one module causes a significant mismatch in feature scale between the deep features in the main branch and the shallow features in the skip branch. Therefore, a dual-attention collaborative mechanism combining the ECA and AFF modules is designed to effectively compensate for the fused feature map shifts. This collaborative approach overcomes the performance limitations of individual attention mechanisms. Furthermore, as the models progress from $Model_{0}$ to $Model_{4}$, the inference time for the forward pass of a single sample gradually increases. By comparing the FLOPs and inference times of $Model_{1}$ and $Model_{3}$, it becomes evident that the AFF module introduces the greatest computational burden. This is because the AFF module contains dual-branch convolutional operations designed to extract global and local attention weights. Although the MFS module also adopts a dual-branch architecture, it remains lightweight overall, with fewer convolutional operations than the AFF module.

Table 6 presents the ablation study results for varying numbers of RAFF modules. As the stacking depth increases, the number of network parameters grows significantly. When the depth reaches four layers, all three evaluation metrics peak, indicating the best recognition performance compared to shallower configurations. Furthermore, we observe that stacking more than four RAFF modules leads to a deep learning degradation problem. In such cases, the recognition accuracy does not improve further and instead declines, while the increased number of parameters results in higher model complexity.

The number of output channels in each RAFF module is a critical hyperparameter, as it directly affects the total number of model parameters. Based on the results in Table 6, the optimal number of stacked layers is determined to be four. We limit the output channel sizes of these four layers to 30 or 60 because the proposed model employs a stacked architecture, where each layer does not require a large number of feature channels. This design enables the network to effectively learn features progressively at each layer. Accordingly, five channel configurations are evaluated. As shown in Table 7, the channel configuration (30, 60, 60, 60) achieves the best recognition performance and offers a balance between complexity and accuracy.

The final set of ablation experiments investigates the impact of the number of parallel atrous convolution branches in the AMAC module. In Table 8, *N* denotes the number of branches, and the dilation rate is a hyperparameter for each atrous convolution branch. The results show that when six atrous convolution branches are used with dilation rates of 1, 3, 5, 7, 9, and 11, the model achieves peak performance across all three evaluation metrics. Although using fewer than six atrous convolution branches reduces the total number of parameters, it causes a performance drop ranging from 3.2% to 9.3% in overall accuracy. Conversely, increasing the number of atrous convolution branches beyond six results in a larger model parameter count without further improvement in recognition performance. Based on these findings, we set the optimal number of atrous convolution branches in the AMAC module to six.

In conclusion, under the optimal configuration, the complete RAFF-AMACNet demonstrates improved and more robust modulation recognition performance while maintaining an acceptable level of model complexity.

## 5. Conclusions

In summary, this paper proposes an automatic modulation recognition method based on the adaptive multi-rate atrous convolution network with residual attentional feature fusion, aiming to address the challenges of modulation recognition under significant Doppler effects and nonlinear distortion in satellite communication systems. First, the multi-scale feature selection module is designed to cascade after multiple atrous convolution branches extract a set of multi-scale features. This module enables the network to adaptively select more effective features from global–local receptive fields, overcoming the limitations of traditional methods that adjust receptive field sizes gradually, layer by layer. Furthermore, a dual-attention collaborative mechanism is integrated into the designed residual modules to enhance the output features of the multi-scale extractor and to correct feature map shifts and accumulated biases caused by significant Doppler frequency shifts and nonlinear effects of satellite-borne equipment during network training. Finally, the pyramid backbone exhibits a simple, sequential architecture that aids the network in aggregating multi-scale features from each layer while producing a deep feature map with reduced dimensionality and greater inter-cluster spacing. Simulation results indicate that this method achieves a recognition accuracy of 92.99% at a low SNR of 0 dB in satellite communication scenarios, where the satellite channel is modeled as a Rician channel with a three-path multipath structure. It is important to note that under more complex and dynamic channel impairments, the recognition performance may degrade to some extent. For example, when the Rician K-factor decreases, the proportion of multipath scattering components increases, and the number of multipath routes rises, the predicted critical SNR for achieving high-accuracy recognition with the proposed model will increase to a value above 0 dB. Our future work will focus on the AMR task for composite modulated signals in satellite communication systems, such as BPSK-PM, QPSK-PM, and BPSK-BPSK-PM. Meanwhile, we will capture real satellite communication signals to investigate the transfer learning mechanism of the network model in practical scenarios.

## Figures and Tables

**Table 1:** The detailed layer-wise parameters of the proposed model.

Layer	Type	Detailed Parameters	Output Size
1	Input	None	(B,2,2048)
2	AMAC	K = 5, S = 1, N = 6, C = 5	(B,30,2048)
3	RAFF1	(ECA):K = 7 (AFF):R = 2	(B,30,1024)
4	RAFF2		(B,60,512)
5	RAFF3		(B,60,256)
6	RAFF4		(B,60,128)
7	GAP	None	(B,60,1)
8	Fc	Out Channel = 11	(B,11)

**Table 2:** Comparison of computational complexity and classification performance across all methods.

Method	Dataset	Params (K)	FLOPs (M)	Inference Time (ms)	OA (%)	Macro-F1 (%)	Kappa (%)
CNN4 [41]	A *	418.21	78.77	0.580	54.16	54.44	49.58
	B *	811.43	155.12	0.520	38.92	39.72	36.27
	C *	1335.72	314.33	0.523	53.79	52.33	49.18
CLDNN [32]	A	1323.78	65.57	0.420	59.00	60.52	54.90
	B	7058.18	524.60	0.882	54.58	57.39	52.61
	C	13,611.78	1049.20	0.920	65.78	65.69	62.37
MCLDNN [25]	A	406.19	49.09	0.240	56.25	56.25	51.88
	B	406.19	402.39	0.243	61.94	63.08	60.29
	C	406.19	806.16	0.237	55.13	52.80	50.65
MCNet [29]	A	184.97	1.41	0.460	53.84	55.08	49.23
	B	184.97	11.24	0.480	41.18	38.65	38.63
	C	184.97	22.55	0.650	57.66	57.20	53.43
ResNet [42]	A	3849.95	21.99	0.405	60.16	61.93	56.18
	B	3849.95	175.92	0.432	57.75	57.75	55.92
	C	3849.95	351.83	0.480	63.74	63.23	60.12
PETCGDNN [44]	A	**72.07**	9.33	**0.014**	59.80	62.19	55.78
	B	**74.95**	71.92	**0.038**	63.00	63.27	61.35
	C	**75.91**	149.28	**0.043**	63.89	63.61	60.28
AWN [43]	A	124.04	12.11	0.391	61.83	64.15	58.01
	B	376.71	140.03	0.468	62.43	62.41	60.76
	C	376.71	279.36	0.507	64.87	64.76	61.36
RAFF-AMACNet	A	123.06	6.87	0.109	**62.51**	**64.86**	**58.76**
	B	123.06	54.71	0.144	**63.90**	**63.98**	**62.33**
	C	123.06	109.39	0.146	**71.74**	**71.55**	**68.91**

* Datasets A, B, and C correspond to RML2016.10A, RML2018.01A, and RML24, respectively.

**Table 3:** Comparison of OA across different SNR levels on the RML24 dataset for all methods.

Method	−18 dB	−12 dB	−6 dB	0 dB	6 dB	12 dB	18 dB
CNN4	11.31	23.77	58.50	73.77	73.59	73.68	73.68
CLDNN	16.31	32.36	65.77	84.90	91.63	97.63	99.04
MCLDNN	11.00	17.81	45.72	70.27	80.40	82.22	82.77
MCNet	**17.36**	27.68	55.99	77.49	85.81	88.27	88.72
ResNet	15.77	29.09	60.18	80.81	89.18	94.22	97.04
PETCGDNN	12.59	23.22	52.45	86.27	93.13	96.04	96.50
AWN	10.41	24.02	55.61	79.19	89.72	97.41	98.66
**RAFF-AMACNet**	15.59	**35.18**	**70.63**	**92.99**	**98.90**	**99.68**	**99.77**

**Table 4:** Three metrics per class for RAFF-AMACNet on the RML24 dataset at an SNR of −4 dB.

Class	Precision (%)	Recall (%)	Macro-F1 (%)
BPSK	1.00	1.00	1.00
QPSK	92.71	94.17	93.44
OQPSK	93.84	95.65	94.73
8PSK	72.00	87.56	79.02
GMSK	99.55	1.00	99.78
16QAM	51.09	33.01	40.11
32QAM	45.26	57.59	50.69
64QAM	47.97	42.41	45.02
SOQPSK-TG	97.39	98.42	97.90
FQPSK	97.87	98.39	98.13
ARTM	98.42	97.41	97.91

**Table 5:** Ablation study of the effectiveness of individual modules on the RML24 dataset.

Model	AMAC (w/o MFS)	AMAC (w/ MFS)	RAFF (ECA)	RAFF (AFF)	Params (K)	FLOPs (M)	Inference Time (ms)	OA (%)
$Model_{0}$	✓	✗	✗	✗	96.91	99.54	0.088	66.51
$Model_{1}$	✓	✓	✗	✗	97.75	99.55	0.109	68.18
$Model_{2}$	✓	✓	✓	✗	97.78	99.89	0.122	68.26
$Model_{3}$	✓	✓	✗	✓	123.04	109.05	0.137	68.69
$Model_{4}$	✓	✓	✓	✓	**123.06**	**109.39**	**0.146**	**71.74**

**Table 6:** Ablation study of the number of RAFF layers on the RML24 dataset.

Number of Layers	Params (K)	FLOPs (M)	OA (%)	Macro-F1	Kappa
1	11.39	30.47	55.20	53.50	50.73
2	41.59	66.79	67.87	67.66	64.66
3	82.33	95.19	70.14	69.88	67.15
**4**	**123.06**	**109.39**	**71.74**	**71.55**	**68.91**
5	163.80	116.50	69.50	69.41	66.45

**Table 7:** Ablation study of the output channels of each layer on the RML24 dataset.

Channels	Params (K)	FLOPs (M)	OA (%)	Macro-F1	Kappa
(30,30,30,30)	43.05	55.96	68.43	68.22	65.28
(30,30,30,60)	62.71	61.41	67.51	67.21	64.27
(30,30,60,60)	92.89	77.41	69.06	68.92	65.97
**(30,60,60,60)**	**123.06**	**109.39**	**71.74**	**71.55**	**68.91**
(60,60,60,60)	153.25	173.36	69.51	69.26	66.46

**Table 8:** Ablation study of the number of atrous convolution branches on the RML24 dataset.

*N*/Dilation Rate	Params (K)	FLOPs (M)	OA (%)	Macro-F1	Kappa
2/(1,3)	14.68	13.31	62.35	64.51	58.59
4/(1,3,5,7)	55.69	49.78	68.51	68.27	65.37
**6/(1,3,5,7,9,11)**	**123.06**	**109.39**	**71.74**	**71.55**	**68.91**
8/(1,3,5,7,9,11,13,15)	216.79	192.15	69.30	69.15	66.23

## Data Availability

In this paper, the RML24, RadioML2016.10A, and RadioML2018.01A datasets are used for experimental validation. The RML24 dataset is the latest dataset for the satellite communication system, while the RadioML2016.10A and RadioML2018.01A datasets serve as representative datasets for testing and evaluating current AMR methods. Readers can obtain these datasets from the author by email (cly@stu.xidian.edu.cn).
